# E-cigarette constituents propylene glycol and vegetable glycerin decrease glucose uptake and its metabolism in airway epithelial cells in vitro

**DOI:** 10.1152/ajplung.00123.2020

**Published:** 2020-09-30

**Authors:** M. Woodall, J. Jacob, K. K. Kalsi, V. Schroeder, E. Davis, B. Kenyon, I. Khan, J. P. Garnett, R. Tarran, D. L. Baines

**Affiliations:** ^1^Institute for Infection and Immunity, St George’s, University of London, Tooting, London, United Kingdom; ^2^Immunology and Respiratory Diseases Research, Boehringer Ingelheim Pharma GmbH & Co. KG, Biberach an der Riss, Germany; ^3^Marsico Lung Institute and Department of Cell Biology and Physiology, University of North Carolina, Chapel Hill, North Carolina

**Keywords:** airway, electronic cigarettes, glucose, glycerin, propylene glycol

## Abstract

Electronic nicotine delivery systems, or e-cigarettes, utilize a liquid solution that normally contains propylene glycol (PG) and vegetable glycerin (VG) to generate vapor and act as a carrier for nicotine and flavorings. Evidence indicated these “carriers” reduced growth and survival of epithelial cells including those of the airway. We hypothesized that 3% PG or PG mixed with VG (3% PG/VG, 55:45) inhibited glucose uptake in human airway epithelial cells as a first step to reducing airway cell survival. Exposure of H441 or human bronchiolar epithelial cells (HBECs) to PG and PG/VG (30–60 min) inhibited glucose uptake and mitochondrial ATP synthesis. PG/VG inhibited glycolysis. PG/VG and mannitol reduced cell volume and height of air-liquid interface cultures. Mannitol, but not PG/VG, increased phosphorylation of p38 MAPK. PG/VG reduced transepithelial electrical resistance, which was associated with increased transepithelial solute permeability. PG/VG decreased fluorescence recovery after photobleaching of green fluorescent protein-linked glucose transporters GLUT1 and GLUT10, indicating that glucose transport function was compromised. Puffing PG/VG vapor onto the apical surface of primary HBECs for 10 min to mimic the effect of e-cigarette smoking also reduced glucose transport. In conclusion, short-term exposure to PG/VG, key components of e-cigarettes, decreased glucose transport and metabolism in airway cells. We propose that this was a result of PG/VG reduced cell volume and membrane fluidity, with further consequences on epithelial barrier function. Taking these results together, we suggest these factors contribute to reduced defensive properties of the epithelium. We propose that repeated/chronic exposure to these agents are likely to contribute to airway damage in e-cigarette users.

## INTRODUCTION

Electronic nicotine delivery systems (ENDS), more commonly known as e-cigarettes, are used to deliver nicotine as an alternative to the use of tobacco. These devices utilize a liquid solution that is drawn over a heating element to produce a vapor, which is then inhaled, giving rise to the term “vaping.” The solutions normally contain the humectants propylene glycol (propane-1,2-diol; PG) and vegetable glycerin (propane-1,2,3-triol; VG) to generate the vapor and act as carriers for nicotine and flavorings. The viscosity of VG is refractory to its sole use in electronic cigarettes. PG and VG are therefore mixed in a wide range of ratios by suppliers of vape products and by end users. Increased PG provides a “throat hit” more like that of cigarette smoking and is thought by some users to improve the carriage of flavorings (20, 43).

There is much debate around the safety of e-cigarettes and the effect of repeated inhalation of these products on the airway. Evidence has indicated that the carriers alone may have cytotoxic effects. Both PG and VG are widely used in the food and cosmetics industry. However, the use of PG as a solvent for drugs delivered intravenously was noted to cause side effects in patients, including lactic acidosis and renal insufficiency (4, 53). Exposure to PG vapor was found to cause upper airway irritation (50), and, following a systematic review of the health effects of e-cigarettes, concern remained regarding the effect of PG on the airway (38). In vitro, PG (at concentrations estimated to be found in the plasma of patients receiving intravenous medication) was shown to have acute cytotoxic and metabolic effects on renal proximal tubule cells (30–33). Furthermore, exposure to PG/VG at a ratio often used in vapes, was found to reduce growth and survival of human embryonic kidney cells and Calu-3 airway epithelial cells (31, 41, 42).

The mechanisms underlying the effects of PG/VG on cell proliferation and viability, particularly in the airway, are unclear. Since glucose uptake is a key metabolic requirement for cell growth and survival and is critical for the normal function of the airway epithelium, we hypothesized that exposure to PG or PG mixed with VG (PG/VG) at a ratio frequently used in vapes would reduce glucose uptake in human airway epithelial cells as a first step to altered cell metabolism.

We therefore investigated the effects of PG and PG/VG (55:45) on glucose uptake in proliferating human H441 and primary bronchial airway epithelial cells (HBECs) in submerged culture and when cultured at the air-liquid interface (ALI) to more closely represent the in vivo airway. We exposed cells to PG or PG/VG applied to the culture medium and vaporized to the apical (luminal) surface of (ALI) cultures. We also explored potential mechanisms determining the action of PG/VG on glucose uptake and metabolism, including osmotic effects and the stability of key glucose transporters known to be expressed in the membrane domain of airway cells by using fluorescence recovery after photobleaching (FRAP).

## MATERIALS AND METHODS

### 

#### Epithelial cell culture.

Human H441 airway epithelial cells (from ATCC, Manassas, VA) were cultured in RPMI 1640 medium containing 10% fetal calf serum (FCS; Invitrogen, UK), 10 mM glucose, 2 mM glutamate, 1 mM sodium pyruvate, 10 μg/mL insulin, 5 μg/mL transferrin, 7 ng/mL sodium selenite, 100 U/mL penicillin, and 100 μg/mL streptomycin. HBECs transduced with human polycomb complex protein BMI-1 (HBEC-BMI-1), were obtained from Prof. S. Hart, Institute of Child Health, University College London, and were cultured as previously described (35). Primary human bronchial epithelial cultures were obtained by the University of North Carolina (UNC) *Cystic Fibrosis* Center Tissue Core under protocols approved by the UNC Institutional Committee for the Protection of the Rights of Human Subjects, as described. H441, BMI1-transduced HBECs, and primary cells were transferred onto clear Transwell (Costar) inserts (1.12 cm^2^ area, 0.45 μm pore size) and grown at air-liquid-interface to form confluent fully differentiated monolayers as described (39, 45, 46). H441 cells were studied 10–14 days postseeding; HBECs were studied 3–5 wk postseeding. Transepithelial electrical resistance (TEER) was measured using an electrovoltometer (EVOM) with chopstick electrodes (WPI UK) and corrected for resistance associated with the Transwell supports. Human embryonic kidney (HEK-293) cells were grown in DMEM + 10% FCS as previously described (42).

PG (3%) or PG/VG (55:45, 3%) was applied to the medium (or directly to the apical (luminal surface) for time periods of 0–24 h, or the cells were exposed to e-cigarette vapor. E-cigarette aerosols were generated using a Sigelei FuChai 200W-TC device with a Crown stainless steel subtank with a 0.25 Ω SUS316 dual coil from Uwell, as previously described in detail (15). We typically generated 70-mL puffs drawn over ∼5 s and dispensed at 0.84 L/min at 100 W. This meant that 20 puffs from our vaping system delivered a concentration equivalent to ∼0.38% e-liquid (vol/vol) per well in 100 μL of PBS or media. Mannitol (7.4% wt/vol was used as an osmotic control in some experiments, which gave a similar osmolarity to that of 3% PG/VG at 408.5 mOsm). All solutions were purchased from Sigma-Aldrich UK.

#### Measurement of cell shrinkage.

For HEK-293T cells, cells were cultured on glass coverslips for 24 h. Epifluorescence measurements were performed using a Nikon Ti-S microscope with Hamamatsu Flash 4.0 camera and Ludl Filter wheels and a ×20 dry plan fluor lens. HBECs were bilaterally loaded with 3 mM calcein-AM (Invitrogen) for 30 min at 37°C (12). Calcein-loaded HBECs were observed by XZ confocal microscopy. Shrinkage was initiated with the mucosal addition of 200 μL of solution (3% PG/VG or mannitol), and cell height and serosal bath intensity were tracked over time (47).

#### HBEC height and confocal airway surface liquid height measurements.

To measure the height of the airway surface liquid (ASL), PBS (20 µL) containing 2 mg/mL rhodamine-dextran (10 kDa; Invitrogen) was added to cultures at the start of the experiment, and all possible fluid was aspirated with a Pasteur pipette to bring ASL volume down to minimal levels. Fifteen predetermined points were automatically XZ scanned using a confocal microscope (Leica SP8; glycerol ×63 immersion lens) as described (9). Cultures were returned to the incubator between time points. For all studies, perfluorocarbon (PFC) was added mucosally during imaging to prevent evaporation of the ASL.

#### Permeability assay.

Culture medium was replaced with Hanks’ balanced salt solution (HBSS; Sigma-Aldrich UK), and cells were incubated with either HBSS alone or with 3% PG/VG in the apical solution. After 30 min, the apical solution was replaced with 0.5 mL of HBSS with 10 µM Na-fluorescein (MW = 367 Da, Sigma-Aldrich). Samples of 0.1 mL were removed from the basolateral bath at 0, 30, 60, and 90 min, and fluorescence was measured in black 96-well plates using a GloMax fluorescence plate reader with excitation and emission wavelengths of 460 nm and 515 nm, respectively.

#### Glucose uptake.

Glucose uptake across the cell membrane was measured using either d-[^14^C]glucose as previously described (23) or using the Uptake Glo luminescent assay (Promega UK). In brief, cultured cells were washed twice with glucose-free transport medium [15 mM HEPES buffer (pH 7.6), 135 mM NaCl, 5 mM KCl, 1.8 mM CaCl_2_, and 0.8 mM MgCl_2_] and then incubated for 15 min to deplete intracellular glucose. Uptake was initiated by replacing with 0.5 mL of transport medium containing 1.0 μCi d-[^14^C]glucose + 10 mM nonradiolabeled d-glucose followed by incubation at room temperature for 10 min. Uptake was terminated by adding 2 mL of ice-cold stop solution [15 mM HEPES buffer (pH 7.6), 135 mM choline Cl, 5 mM KCl, 0.8 mM MgSO4, 1.8 mM CaCl_2_, and 0.2 mM HgCl_2_]. The cells were rinsed twice with stop solution and lysed in 0.5 mL of 10 mM Tris.HCl (pH 8.0) + 0.2% SDS. Lysed samples were added to 2 mL of scintillation cocktail, and radioactive emissions were determined using a scintillation counter to quantify glucose uptake. For the Uptake Glo luminescent assay, uptake was initiated using 2-deoxyglucose (2-DG) solution per the manufacturer’s protocol. Samples were similarly incubated for 10 min before addition of stop buffer. After neutralization, the detection reagent was added, and luminescence was recorded with 1-s integration. For both protocols, all steps took place in the presence or absence of PG (3%), PG:VG (55:45, 3%), mannitol (7.4%), and/or glucose transport inhibitors phloretin (1 mM dissolved in ethanol) and cytochalasin B (10 μM) or the aquaporin 3 inhibitor (DPF00173, 50 μM). Data are presented as percent control to normalize between protocols.

#### Seahorse assay.

Human bronchiolar epithelial cells from two independent donors per experiment were seeded into a Seahorse XF96 plate and incubated at 37°C, 5% CO_2_ for 48 h. The medium was changed 24 h before Seahorse experiment, and cells were exposed to PG:VG (55:45, 3%) and mannitol (7.4%) for 30 min before a Seahorse glycolysis stress assay was performed according to the manufacturer’s instructions, followed by the sequential injection of oligomycin (1 μM) to inhibit ATP-linked respiration and 2-DG (50 mM) to inhibit glucose metabolism. The plate layout was separated into quadrants to reduce edge effects. Extracellular acidification rate (ECAR) and oxygen consumption rate (OCAR) were measured. Glycolysis rate was calculated by subtracting ECAR values after 2-DG injection from the ECAR values after glucose injection to exclude the nonglycolytic acidification from the calculation. Glycolytic capacity was calculated by subtracting the nonglycolytic acidification rate (ECAR after 2-DG injection) from the maximum ECAR after 1 μM oligomycin injection.

#### Fluorescence recovery after photobleaching.

Fluorescence recovery after photobleaching (FRAP) was carried out on human embryonic kidney cells transfected with GLUT1-GFP, GLUT2-mCherry, or GLUT10-GFP. HEK-293T cells were seeded on no. 1.5 glass coverslips and transiently transfected with constructs, using Lipofectamine 2000 (Thermo Fisher) per the manufacturer’s instructions. Fluorescence recovery after photobleaching was performed 24–48 h after transfection, using a Leica SP5 confocal microscope with a 63 × 1.30 numerical aperture glycerol immersion lens as described (25).

#### Western blots: phosphorylated mitogen-activated protein kinase and quantification.

Cells were lysed in lysis buffer (50 mM Tris.HCl, pH 7.4, 1% NP-40, 0.25% sodium deoxycholate, 150 mM NaCl, 1 mM EDTA, 1 mM EGTA, 1 mM PMSF, 1 mM Na_3_VO_4_, 50 mM NaF, 5 mM sodium pyrophosphate, and 1% (wt/vol) protease inhibitor cocktail). Cellular debris was removed by centrifugation at 13,000 *g* for 10 min, and protein concentrations were determined using the Bradford assay (as above). Approximately 30–50 µg of proteins was fractionated on 4–12% bis-tris gel (Invitrogen, Paisley, UK) alongside prestained protein standards (Santa Cruz, CA) and transferred onto Hybond-P PVDF Membrane (GE Healthcare, Amersham, UK). Membranes were incubated in TBS-T containing 5% (wt/vol) nonfat milk powder for 1 h at room temperature before incubation with primary antibodies; anti-phospho-p38 mitogen-activated protein kinase (MAPK; Thr^180^/Tyr^182^) Antibody no. 9211 or anti-p38 MAPK no. 9212 (Cell Signaling Technology) (all diluted 1:1,000) followed by incubation with species-specific horseradish peroxidase-conjugated secondary antisera (Sigma UK).

Immunostained proteins were visualized with SuperSignal West Pico chemiluminescent substrate (GE Healthcare, Amersham, UK). Densitometric quantification was performed using Scion Image (NIH, Bethesda, MD).

#### Cytotoxicity.

Cytotoxicity was measured by analyzing lactate dehydrogenase (LDH) release from cells using an LDH cytotoxicity assay (Pierce UK), and cell proliferation was analyzed using the CyQuant Direct Cell Proliferation Assay according to the manufacturer’s instructions. Assays were carried out 30 min after exposure to treatments as described above.

#### Statistical analysis.

For statistical analysis, *P* ≤ 0.05 was taken as significant. Data were analyzed by ANOVA with post hoc Tukey/Bonferroni test where indicated. Data are shown as means ± SE, where *n* = the number of independent experiments (cell lines) or replicates/measurements from primary cells where the number of donors are stated.

## RESULTS

### 

#### PG and PG/VG inhibit glucose transport.

**Fig. 1.**
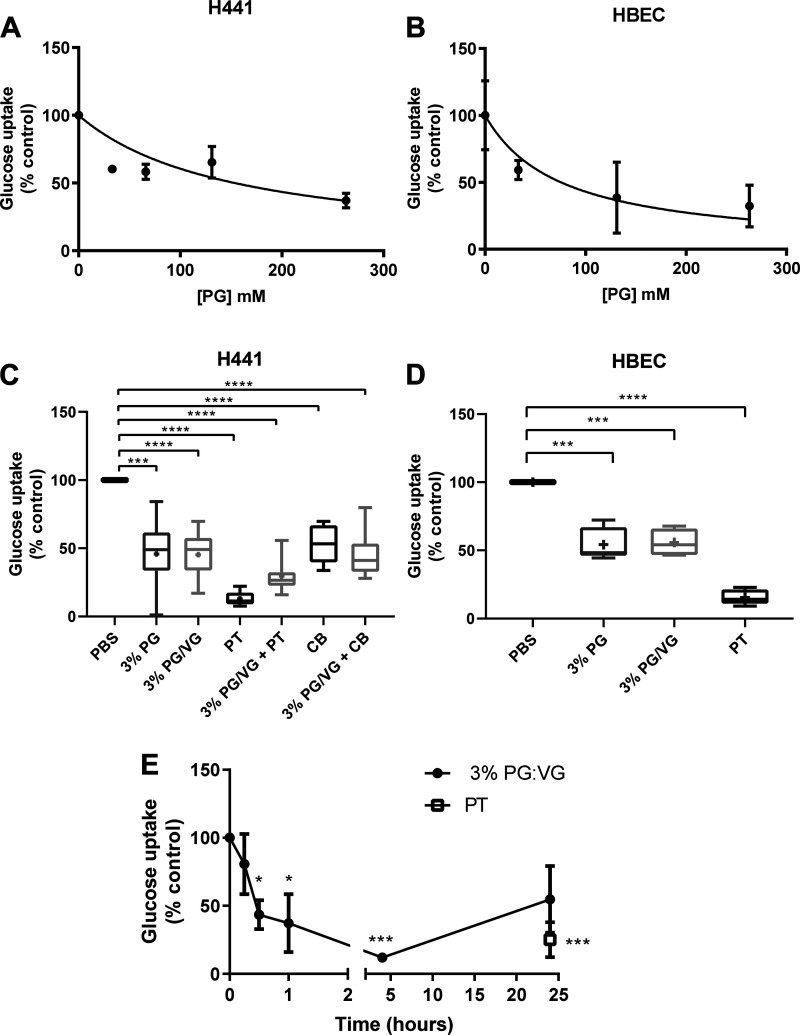
Propylene glycol (PG) and PG mixed with vegetable glycerin (PG/VG) inhibit glucose uptake in proliferating airway cells. Exposure to PG (33–263 mM) for 30 min decreased glucose uptake, shown as %control in a concentration-dependent manner in H441 (*A*) and BMI-1-transduced (*B*) human bronchiolar epithelial cells (HBECs). Inhibition of glucose uptake after 30-min exposure to 3% PG (263 mM) or 3% PG/VG (45:55) with and without GLUT inhibitors phloretin (PT) and cytochalasin B (CB), or PT or CB alone in H441 (*C*) or BMI-1-transduced (*D*) HBECs. *E*: exposure of H441 cells to 3% PG/VG (45:55, closed circles) decreased glucose uptake in a time-dependent manner. The effect of PT (open squares) on glucose transport at 24 h is shown for reference. Data are shown as box and whisker plots. Horizontal line, median; box, 25–75th percentiles; whiskers, min and max; +, mean. Significantly different from control: *P < 0.05, **P < 0.01, ***P < 0.001, and ****P < 0.0001.

In airway cells, the primary route for glucose uptake is via glucose transporters (23). Glucose transport is often higher in proliferating cells to support cellular growth and particularly in immortalized cell lines such as H441 airway cells. Furthermore, when airway cells are grown at air-liquid interface, glucose transporters are differentially segregated to apical and basolateral domains (22, 29, 37). Therefore, we first investigated the effect of exposure to PG on glucose uptake in proliferating H441 cells grown on plastic. PG inhibited glucose uptake in a dose-dependent manner with 263 mM, (equivalent to 3% PG wt/vol), inhibiting glucose uptake to less than 50% of control (Fig. 1, *A *and *B*).

We then investigated the effect of exposure to 3% PG and 3% PG/VG (55:45, a common mix in electronic cigarettes) for 15 min in both proliferating H441 and BMI-1-transduced HBECs (which have extended passage potential while retaining the characteristics of primary cells). Three percent PG and 3% PG/VG inhibited glucose uptake in proliferating H441 and BMI-1-transduced HBECs to 46 ± 23 and 45 ± 17%, respectively, of control (*P* < 0.001, *n* = 18). Phloretin, which blocks GLUT-mediated glucose transport had a more potent effect, reducing glucose uptake to 13 ± 1% of control (23). The effect of 3% PG/VG was not additive to that of phloretin (Fig. 1*C*). In addition, the effect of 3% PG/VG was not additive to the effect of an alternative glucose transport inhibitor cytochalasin B (10 μM). Glucose transport was reduced to 54 ± 13 and 45 ± 15%, *n* = 12, respectively (Fig. 1*C*). These data indicate that PG/VG inhibited glucose uptake via glucose transporters.

PG at 3%, 3% PG/VG, and phloretin had a similar effect on proliferating BMI-1-transduced HBECs, reducing glucose uptake to 54 ± 12, 56 ± 10, and 15 ± 5%, respectively (*P* < 0.001, *n* = 6; Fig. 1*D*).

Exposure to 3% PG/VG reduced glucose transport maximally at 4 h, reaching levels that were similar to that of the glucose transport inhibitor phloretin. However, glucose transport recovered to ∼50% of control at 24 h. (Fig. 1*E*)

Glucose transport is one of the rate-limiting steps of intracellular glucose metabolism; therefore, we investigated the effect of PG and PG/VG on glycolysis and mitochondrial ATP generation. Since exposure to PG and PG/VG can elicit osmotic effects (31), we also compared their effect to that of exposure to 7.4% mannitol, which was isoosmolar with PG/VG, with a calculated osmolarity of 408.5 mOsm.

#### PG and PG/VG modify airway cell glucose metabolism.

**Fig. 2.**
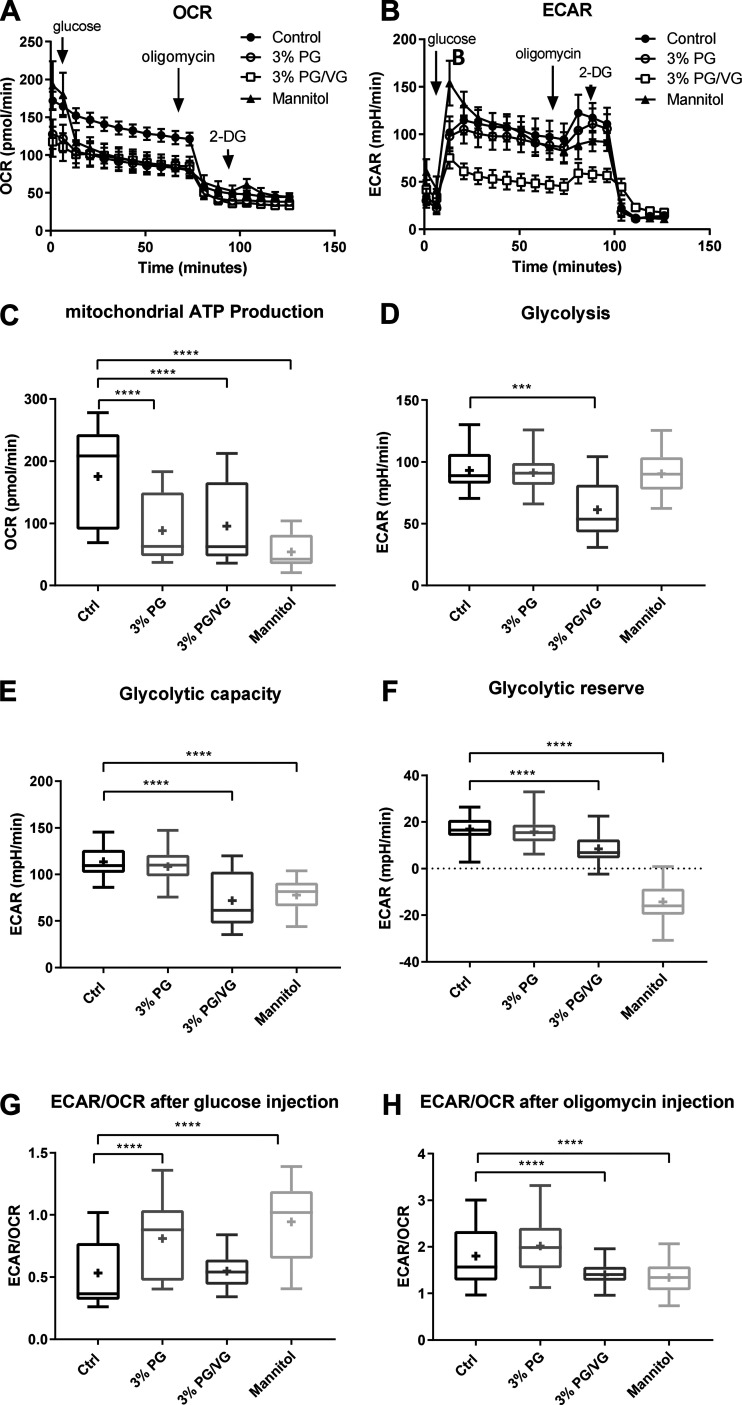
Propylene glycol (PG) and PG mixed with vegetable glycerin (PG/VG) modify airway cell glucose metabolism. The effect of 3% PG or 3% PG/VG, 45:55 or isosmotic mannitol on human bronchiolar epithelial cell (HBEC) metabolism. A. Oxygen consumption rate (OCR; *A*) and extracellular acidification rate (ECAR; *B*) after sequential addition of glucose, oligomycin, and 2-deoxy-d-glucose (2-DG). *C*: mitochondrial ATP production measured as OCR. Glycolysis (D), glycolytic capacity (*E*), and glycolytic reserve (*F*) all measured as ECAR. *G*: ECAR/OCR after glucose injection to initiate metabolism. *H*: ECAR/OCR after oligomycin injection to inhibit oxidative phosphorylation. Data are shown as box and whisker plots. Horizontal line, median; box, 25–75th percentiles; whiskers, min and max; +, mean. Significantly different from control: ***P < 0.001 and ****P < 0.0001.

**Fig. 3.**
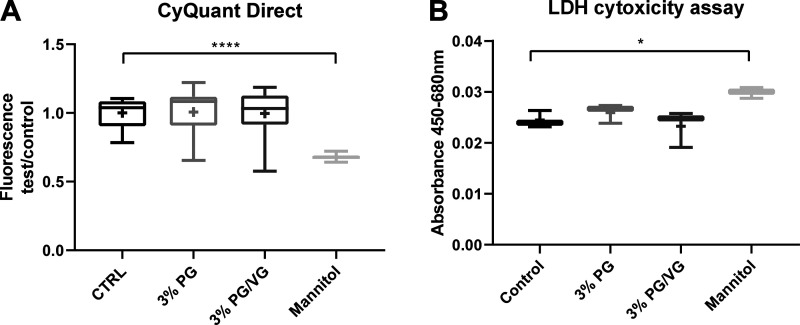
Propylene glycol (PG) and PG mixed with vegetable glycerin (PG/VG) did not inhibit airway cell proliferation or elevate cytotoxic markers. Effect of 30-min exposure to 3% PG or 3% PG/VG, 45:55, or isoosmotic mannitol on human bronchiolar epithelial cells (HBECs). *A*: CyQuant Cell Proliferation Assay of DNA content measured as fluorescence. *B*: lactate dehydrogenase (LDH) release measured as absorbance at 450–680 nm. Data are shown as box and whisker plots. Horizontal line, median; box, 25–75th percentiles; whiskers, min and max; +, mean. Significantly different from control: *P < 0.05 and ****P < 0.0001.

Having demonstrated that PG and PG/VG reduced glucose uptake in BMI-1-transduced HBECs, we then used primary HBECs, from a total of four independent donors, to investigate effects on metabolism by measuring OCR; OCR and extracellular acidification rate; ECAR after the sequential addition of d-glucose (10 mM), oligomycin (1 μM) and 2-DG (50 mM; Fig. 2, *A *and* B*). PG at 3%, 3% PG/VG and isoosmotic mannitol all significantly reduced mitochondrial ATP generation (measured as OCR) in these airway cells compared with control (*P* < 0.001, *n* = 72, 71, and 66 replicates, respectively; Fig. 2*C*). Compared with control, PG/VG and mannitol, but not PG, reduced glycolytic capacity (*P* < 0.0001, *n* = 71 and 66 replicates, respectively) and glycolytic reserve (*P* < 0.0001, *n* = 66) (Fig. 2, *E *and* F*). Only PG/VG inhibited glycolysis (*P* < 0.001, *n* = 71). This indirect evidence raised the possibility that VG mediated the inhibition of glycolysis (Fig. 2*D*). ECAR/OCR after glucose or oligomycin injection reflected the differential effects of PG, PV/VG, and mannitol on glycolytic parameters and mitochondrial ATP generation (Fig. 2, *G *and* H*). There was no effect of exposure to 3% PG and 3% PG/VG on cell proliferation or lactate dehydrogenase release, indicating no initial toxicity. However, exposure to isoosmolar mannitol significantly reduced cell proliferation to 68 ± 2% of control (*P* < 0.0001, *n* = 11 replicates) and increased LDH release to 122 ± 1% compared with control (*P* < 0.05, *n* = 3 replicates) (Fig. 3, *A *and* B*).

#### PG/VG reduced cell surface area.

**Fig. 4.**
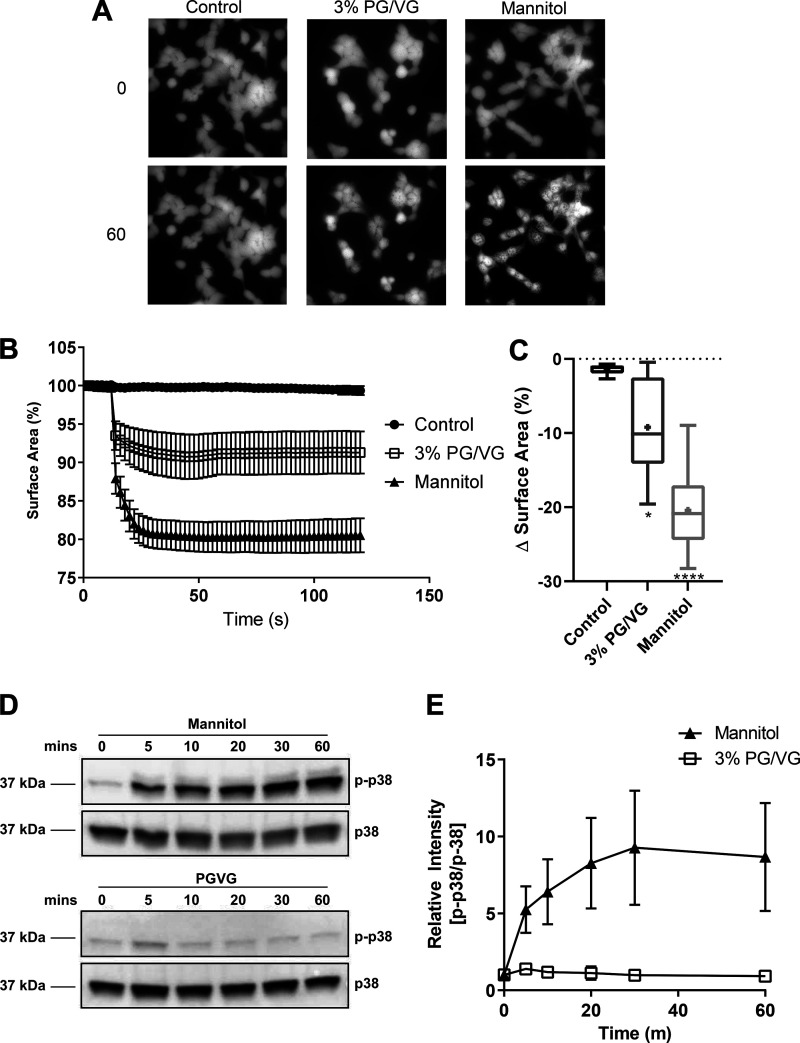
Propylene glycol (PG) and PG mixed with vegetable glycerin (PG/VG) decreases cell surface area but does not elevate p38 mitogen-activated protein kinse (MAPK). *A*: fluorescence microscopy of calcein-AM-loaded HEK-293 cells before (control) and after exposure to 3% PG/VG or isoosmotic mannitol. Calcein-fluorescent cells are seen as bright white in images. *B*: surface area (%), of cells exposed to 3% PG/VG or mannitol (as shown in *A*) over a time course of 120 s. *C*: collated data of percent change in surface area (ΔSurface Area, %) as shown in *B*. Data are shown as box and whisker plots. Horizontal line, median; box, 25–75th percentiles; whiskers, min and max; +, mean. *D*: Western blots of phosphorylated p38 MAPK (p-p38, *top*) and nonphosphorylated p38 MAPK after exposure to mannitol or 3% PG/VG for 0–60 min. *E*: quantification of p-p38/total p38MAPK from *n* = 3 experiments. Data are shown as means ± SD.

To examine effects on cell surface area, we used HEK-293 epithelial cells because of their size and suitability for imaging. Exposure to 3% PG/VG or isoosmolar mannitol rapidly (within 20 s) reduced cell surface area (by −9.2 ± 2.4 and −20.4 ± 2.3%, respectively, compared with control; −1.4 ± 0.3%, *P* < 0.01 and 0.0001, *n* = 7), indicating cell shrinkage consistent with a hyperosmotic effect (Fig. 4, *A *and* B*). Phosphorylation of p38 MAPK, which is associated with osmotic stress, was elevated only after exposure to mannitol (reaching maximum at 30 min, *P* < 0.0001, *F*(1, 24) = 24.69) and not PG/VG, consistent with its more potent effect (Fig. 4, *C *and* D*). These data indicate that the effect of PG/VG on glucose transport and metabolism is not via activation of p38 MAPK.

#### PG/VG reduced airway epithelial height, decreased transepithelial resistance, and increased epithelial permeability and ASL height.

**Fig. 5.**
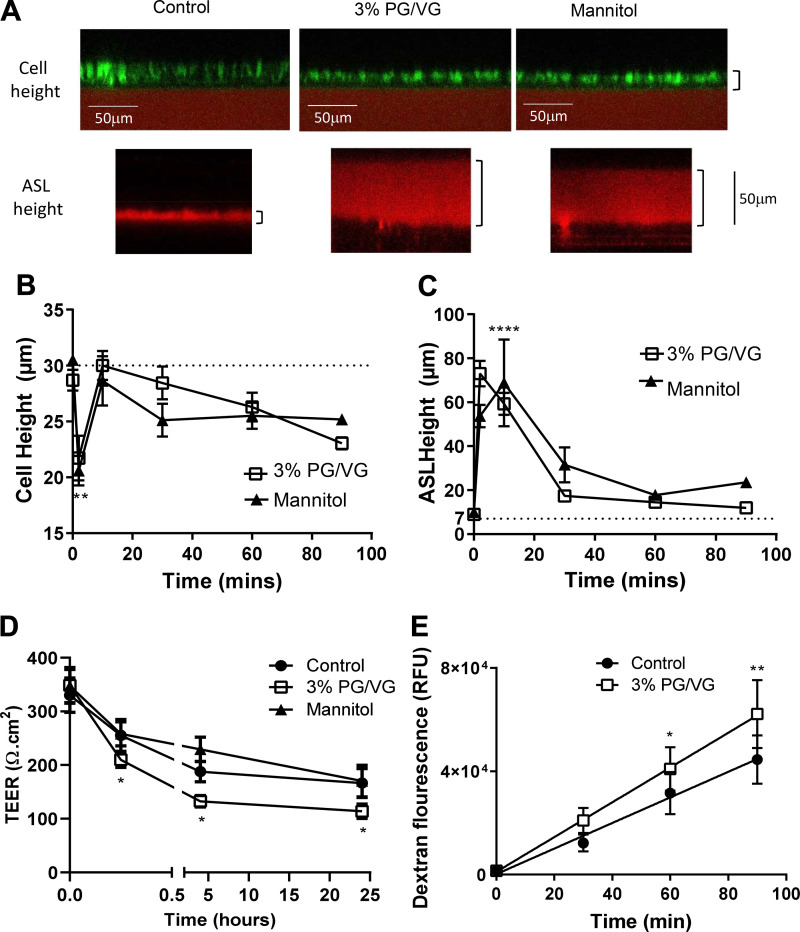
Propylene glycol mixed with vegetable glycerin (PG/VG) reduced airway epithelial height, decreased transepithelial resistance, and increased epithelial permeability and airway surface liquid (ASL) height *A*: representative fluorescence microscopy images of calcein-AM-loaded human bronchiolar epithelial cells (HBECs) (green; for cell height measurement) or rhodamine-dextran-labeled airway surface liquid (ASL) overlying HBECs (red; for ASL height measurement) before (control) and after mucosal exposure to 3% PG/VG or mannitol. Effect of control (closed circles), 3% PG/VG (open squares) or isoosmotic mannitol (closed triangles) on cell height (*B*) and ASL height (*C*). Transepithelial electrical resistance (TEER; *D*) and transepithelial permeability (*E*) measured across H441 cells grown at air-liquid interface. Dotted lines in *B *and* C* represent untreated control levels. RFU, relative fluorescence units. Data are shown as means ± SD. Significantly different from control: *P < 0.05, **P < 0.01, and ****P < 0.0001.

To better model the in vivo airway, we investigated the effect of mucosal PG/VG on differentiated HBECs grown at air-liquid-interface on permeable supports. Apical application of 3% PG/VG and mannitol initiated a rapid reduction in HBEC cell height from 28.7 ± 2.9 and 30.5 ± 1.5 μm to 21.7 ± 5.7 and 20.6 ± 4.2 μm, respectively (*P* < 0.001, *n* = 10 measurements from 3 independent donors). Cell height initially recovered at ∼10 min but then exhibited a continual decline to 90 min (Fig. 5, *A *and* B*). Consistent with an apical osmotic challenge, airway surface liquid height rapidly increased from 9 ± 2 to 73 ± 18 and 54 ± 16, *P* < 0.001, *n* = 10, respectively, after 2 min and remained elevated up to 10 min. ASL then slowly declined but did not reach initial levels even after 90 min (Fig. 5*C*). In parallel, apical exposure to 3% PG/VG resulted in a greater decrease in HBEC TEER from 1,142 ± 433 to 685 ± 309 Ω.cm^2^ than cells exposed to apical isoosmotic mannitol 920 ± 169 Ω.cm^2^ (*P* < 0.05, *n* = 4 independent donors). Repeated measurement of TEER causes a decrease over time. However, similar apical exposure of H441 cells to 3% PG/VG resulted in a more rapid and greater decrease in TEER than in cells exposed to apical isoosmotic mannitol (0–15 min, from 348 ± 33 to 209 ± 22 and 347 ± 31 to 258 ± 22 Ω.cm^2^, respectively, *P* < 0.05, *n* = 15). The additional reduction in TEER was sustained over a period of 24 h (Fig. 5*D*). The decrease in resistance correlated with an increase in apical to basolateral permeability of the epithelial monolayer to Na^+^ fluorescein measured over 90 min (Fig. 5*E*). These data indicate that PG/VG induces effects on cell volume consistent with hyperosmotic effects and additional detrimental effects on airway epithelial barrier function.

#### PG/VG decreases FRAP of glucose transporters in the membrane.

**Fig. 6.**
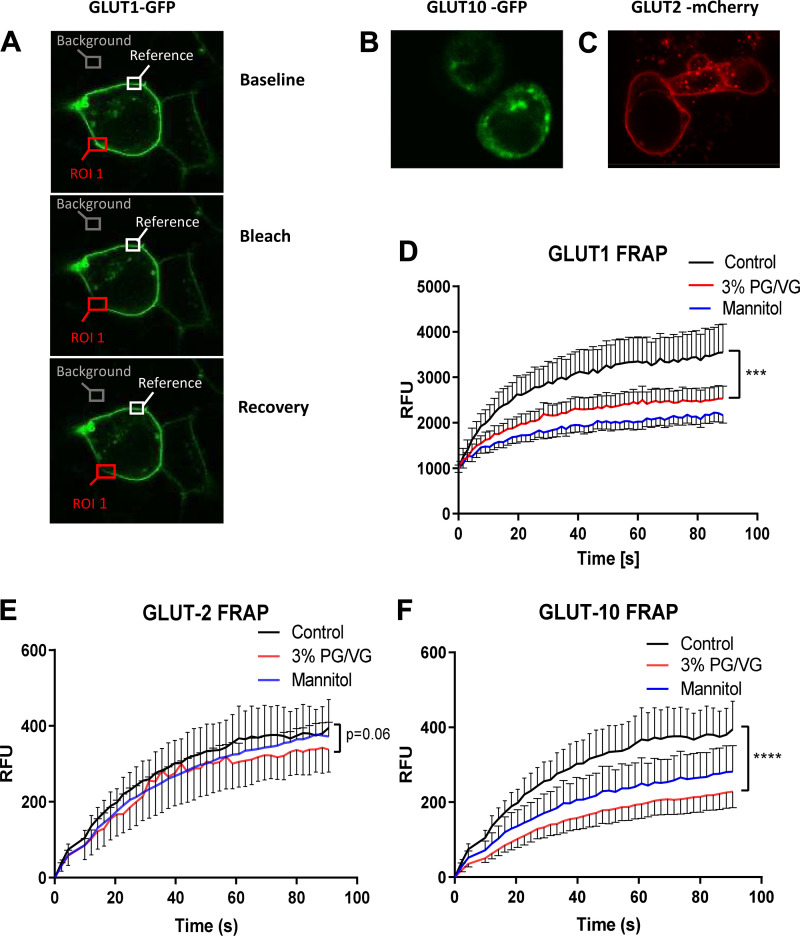
Propylene glycol mixed with vegetable glycerin (PG/VG) decreases fluorescence recovery after photobleaching (FRAP) of glucose transporters. *A*: fluorescence of GLUT1-green fluorescent protein (GFP) expressed in HEK-293 cells showing areas measured for background, membrane fluorescence and region of interest (ROI) at baseline, after fluorescent photobleaching (Bleach), and recovery. GLUT10-GFP (*B*) and GLUT2-mCherry (*C*) expressed in HEK-293 cells. Graphs showing FRAP in control cells (black line) or after treatment with 3% PG/VG (red line) or mannitol (blue line) over a time course of 90 s for GLUT1-GFP (*D*), GLUT2-mCherry (*E*), and GLUT10-GFP (*F*). RFU, relative fluorescence units. Data are shown as means ± SE, with error bars shown in one direction for clarity. Significantly different from control: as shown or ***P < 0.01 and ****P < 0.0001.

We then investigated the effect of 3% PG/VG and mannitol on the turnover/movement of glucose transporters in the cell membrane by use of fluorescence recovery after photobleaching in HEK-293 cells. We investigated GLUT1, -2, and -10, which have all been proposed to play a role in glucose uptake in airway cells. Exposure to 3% PG/VG or mannitol significantly reduced recovery of fluorescence of GLUT1-GFP and GLUT10-GFP expressed in the membrane domain compared with control (*P* < 0.001, *n* = 10 independent cell recordings). FRAP of GLUT2-mCherry was less affected by either 3% PG/VG or mannitol. These data indicate that exposure to PG/VG and mannitol reduce glucose uptake in airway cells via mechanisms that reduce the turnover and function of glucose transporters at the membrane (Fig. 6, *A*–*F*).

#### PG/VG vaped onto the apical surface of HBECs decreases glucose transport.

**Fig. 7.**
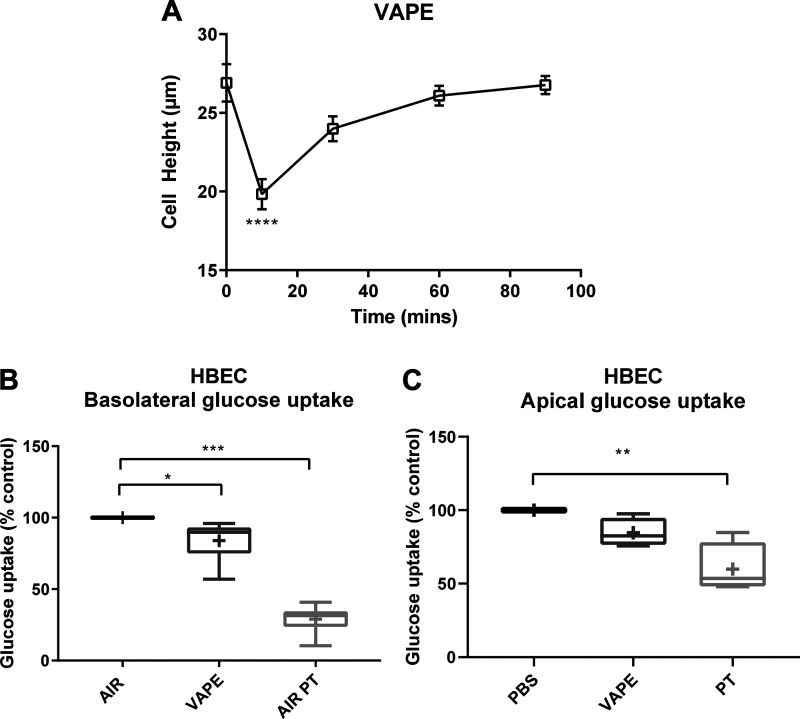
Propylene glycol mixed with vegetable glycerin (PG/VG) vaped onto the apical surface of human bronchiolar epithelial cells (HBECs) decreases glucose transport. Effect of 3% PG/VG puffed onto mucosal surface (VAPE) of primary HBECs on cell height: effect of 3% PG/VG (VAPE) (*A*), or air (AIR) puffed onto mucosal surface or air plus the addition of phloretin (AIR PT) on glucose uptake across the basolateral (serosal) membrane (*B*) and glucose uptake across the apical (mucosal) membrane (*C*). Data are shown as box and whisker plots. Horizontal line, median; box, 25–75th percentiles; whiskers, min and max; +, mean. Significantly different from control as shown or *P < 0.05, **P < 0.01, and ***P < 0.001.

Finally, to more physiologically mimic the way e-cigarette vapor reaches the epithelium, we investigated the effect of 3% PG/VG vaped/puffed onto the apical surface of differentiated HBECs grown at ALI at 27 puffs/10 min on cell height. The reduction of cell height was slower but of similar magnitude (from 27 ± 5 to 20 ± 4 μm; Fig. 7*A*) to that after direct application of PG/VG (see Fig. 5*B*).

A similar treatment of 3% PG/VG puffed onto the apical surface of HBECs mildly reduced basolateral glucose uptake to 84 ± 5% compared with puffing with air alone (*P* < 0.05, *n* = 8 from 4 independent donors). Air with basolaterally applied phloretin inhibited to 29 ± 3% of control, consistent with previous observations. The effect of 3% PG/VG on apical glucose uptake was not statistically significant. Phloretin inhibited apical glucose uptake to only 60 ± 9% of control (*P* < 0.05, *n* = 4 from 4 independent donors).

## DISCUSSION

There is increasing evidence that exposure to e-cigarette vapor induces toxicity, oxidative stress, and inflammatory responses in lung epithelial and endothelial cells (26). Toxicity associated with flavorings have been described (19, 40, 42), as have the effects of nicotine (11, 13). However, less is known about the effects of PG and PG/VG. Since it was reported that PG/VG reduced cell growth (19, 42), we set out to investigate whether PG and PG/VG affected glucose uptake and metabolism in airway cells as a potential mechanism for growth inhibition.

Our data are the first to show that short-term exposure to PG or PG/VG at concentrations comparable to those used in in vitro studies as a model of lung exposure (3% wt/vol) inhibited glucose uptake and suppressed mitochondrial ATP production in airway cells. The dose dependency of PG on glucose uptake and metabolism was consistent with that described in renal cells (31–34). Similarly to our observations, PG was shown to inhibit Na^+^-independent, GLUT-mediated glucose uptake in renal cells. We also found evidence that 3% PG/VG inhibited glycolysis. As this was not observed with PG only, it is possible that VG mediated this effect. Further work is now required to see if this is indeed the case. Importantly, even in the context of a single exposure to e-cigarette vapor, a decrease in glucose uptake across the serosal membrane highlighted the potency and ability of PG/VG to affect even compartmentalized function of the airway epithelium.

The mechanisms underlying the effects we observed are complex. The addition of sugar alcohols/polyols increases the osmolality of solutions. In this study, we maintained the osmolality of the mannitol, PG, and PG/VG solutions at ∼408 mOsm (hyperosmotic). Our finding that these solutions induced rapid changes to HEK-293 cell surface area, cell height, and ASL height in HBECs indicate hyperosmotic effects on cell volume and a potential link to reduced glucose uptake and metabolism. Such pathways have been reported. Hyperosmotic stress was proposed to decrease ATP synthesis in HT22 nerve cells (14), and exposure to hyperosmotic mannitol in the rat jejenum decreased glucose uptake (26).

Mannitol [which inhaled is used to test airway hyperresponsiveness (7)] also induced a potent phosphorylation of p38 MAPK, typical of classical signaling in osmotic stress (8, 16, 54). Phosphorylation of p38 MAPK in response to osmotic stress has previously been demonstrated in both HEK-293 and human airway cells (54). Phosphorylation of p38 MAPK regulates the gene expression of key cellular processes such as proliferation and apoptosis. Activation of p38 MAPK was also important for rebalancing the cells’ energy status, and post-osmotic insult, by induction of GLUT1 expression in cardiomyocytes and rat liver Clone 9 cells, but only after more prolonged exposure (12 h) (10, 16, 21, 44).

Surprisingly, PG/VG did not activate p38 MAPK [and exposure of airway cells previously to PG/VG did not elicit a rise in intracellular Ca^2+^ (42)]. We have not yet investigated activation of other signaling pathways [e.g., AMPK, which induced glucose uptake in Clone 9 cells, preadipocytes, and myoblasts (1)]. Nevertheless, the ability of PG/VG to induce similar cell volume changes without inducing phosphorylation of p38 MAPK raises the possibility that adaptation to PG/VG insult is different and could have consequences for cell survival. Certainly, over a longer time course, 3% PG/VG was reported to reduce the viability of airway cells (41, 42).

In previous studies, 3% PG/VG produced a decrease in the fluorescence of merocyanine 540 (M540) incorporated into the plasma membrane of HBECs, indicating an increase in molecular packing stress and reduction of membrane fluidity (19, 25). Mannitol-induced cell shrinkage would also result in increased surface packing of membrane lipids and a reduction in membrane fluidity. Such a reduction in membrane fluidity would provide a mechanism for the reduced recovery of facilitative glucose transporters that we observed in the membranes of airway cells after photobleaching. In particular, FRAP of GLUT1 and GLUT10 were potently reduced. GLUT1 is ubiquitous and the primary route for glucose uptake in proliferating cells, and it has been demonstrated on the basolateral membrane of differentiated HBEC (37). GLUT10 has been observed in the apical membrane domain of H441 cells and HBECs, but its role in glucose uptake in the airway is not well understood, and it may have additional roles as a mitochondrial transporter (2, 3, 17, 22, 23). Both transporters have a higher affinity for glucose than GLUT2, which was modestly affected (*K*_m_ ∼3, ∼0.3, and 17 mM, respectively). Thus, under normal culture and experimental conditions, where glucose concentration is 5–10 mM, although we cannot rule out competitive inhibition of glucose transport by PG or PG/VG, the effect of membrane packing stress and reduced diffusibility of GLUT1 and -10 would likely suppress glucose transport and consequently glucose metabolism during the hyperosmotic insult.

PG/VG also induced a rapid decrease in TEER in H441 airway cells, but mannitol did not. Interestingly, mannitol did not cause a decrease in TEER in differentiated 16HBE14o- airway cells when osmolarity was below 450 mOsm (36). Unlike mannitol, PG and VG can permeate membranes, and high VG concentration was shown to modify membrane structure not only by osmotic volume contraction but also by direct interactions with the lipid components in the membrane (5, 6, 27). Such interaction with the plasma membrane is likely to produce detrimental effects on the tight junctions between airway epithelial cells, causing the decrease in TEER and subsequent increase in transepithelial permeability. In support of this notion, glycerol was shown to disrupt tight junction-associated proteins F-actin, occludin, and tubulin organization in rat Sertoli cells. A derivative of PG, propylene glycol caprylate, also decreased intestinal epithelial TEER via effects on tight-junction proteins, including occludin and zonula occludens-1, which are present in airway cells (18, 24, 48, 49). We did not investigate effects on ASL height beyond 90 min, but a sustained loss of barrier function, as seen in H441 cells, would likely result in an inability to regulate airway surface liquid volume and disrupt innate immune function of the airway and predispose to infection (27, 28, 51, 52).

In conclusion, we show that short-term exposure to PG and VG, key components of e-cigarettes, either in solution or vaped on the luminal surface of airway cells, decreased GLUT-mediated glucose transport and ATP production in airway cells. We propose that this occurred as a result of changes to cell volume and membrane fluidity caused by osmotic effects and direct interaction of PG/VG with lipids in the cell membrane. PG/VG also decreased barrier function and increased epithelial permeability. Taking all these together, we suggest that all these factors contribute to reduced defensive properties of the epithelium, which could lead to increased susceptibility of the lungs to infection. We propose that repeated/chronic exposure to these agents is likely to contribute to airway damage in e-cigarette users.

## GRANTS

M.W. was funded with a The Cystic Fibrosis Trust studentship; J.J. was funded by St George’s, University of London; K.K.K. was funded by Astra Zeneca, Gothenburg, Sweden. J.P.G. was funded by a Respiratory Diseases Research Award from the Medical Research Foundation (MRF-091-0001-RG-GARNE), and R.T. was funded by National Heart, Lung, and Blood Institute Grant HL135642.

## DISCLOSURES

No conflicts of interest, financial or otherwise, are declared by the authors.

## AUTHOR CONTRIBUTIONS

J.P.G., R.T., and D.L.B. conceived and designed research; M.W., J.J., K.K.K., V.S., E.D., B.K., I.K., J.P.G., R.T., and D.L.B. performed experiments; M.W., J.J., K.K.K., V.S., E.D., B.K., I.K., J.P.G., R.T., and D.L.B. analyzed data; J.J., K.K.K., E.D., B.K., J.P.G., R.T., and D.L.B. interpreted results of experiments; M.W., J.J., K.K.K., E.D., B.K., J.P.G., R.T., and D.L.B. prepared figures; D.L.B. drafted manuscript; P.G., R.T., and D.L.B. edited and revised manuscript; M.W., J. J., J.P.G., R.T., and D.L.B. approved final version of manuscript.
